# Increasing Prevalence of Pediatric Type 2 Diabetes in the Republic of Ireland: A National Cross-Sectional Study

**DOI:** 10.1155/pedi/8892271

**Published:** 2025-09-11

**Authors:** Katie Lynam, Michael J. O'Grady

**Affiliations:** ^1^Department of Paediatrics, Regional Hospital Mullingar, Co. Westmeath, Ireland; ^2^Women's and Children's Health, School of Medicine, University College Dublin, Dublin 4, Ireland

**Keywords:** adolescent, child, obesity, prevalence, type 2 diabetes mellitus

## Abstract

**Aims:** To establish the current prevalence of type 2 diabetes in children and adolescents aged under 16 years in the Republic of Ireland, to identify modes of presentation, patient characteristics, comorbidities, management, and outcomes.

**Methods:** We conducted a cross-sectional study of children and adolescents aged under 16 years with a diagnosis of type 2 diabetes in September 2023 using a standardized proforma. This was circulated to all clinicians providing care to children with diabetes in all 19 centers in the Republic of Ireland.

**Results:** Thirty-two cases of type 2 diabetes were identified, giving an estimated prevalence in children and adolescents under 16 years of 3/100,000 population, a significant increase from 1.2/100,000 population in 2015 (*p*  < 0.004). This was due to increased prevalence rates in, both White and Asian populations, as well as an increase in the size of the Asian population under 16. Nineteen (59%) were girls. Median duration of diabetes was 1.2 (0.1–4.9) years. Median body mass index (BMI) *z*-score at diagnosis was identical to the 2015 study (+2.3). Sixteen (50%) achieved the target HbA1c specified by the International Society for Pediatric and Adolescent Diabetes (ISPAD) of 48 mmol/mol (6.5%) or less. Completion of screening for comorbidities and complications of type 2 diabetes were not in accordance with guidelines.

**Conclusion:** There has been a significant increase in the prevalence of type 2 diabetes in under 16's in a short timeframe. Establishment of a National Diabetes Register will facilitate ongoing monitoring of disease epidemiology in this and other age cohorts.

**Summary**

• **What is already known?**
  • A previous study in the Republic of Ireland in 2015 established a prevalence of type 2 diabetes in under 16's of 1.2/100,000.
• **What this study has found?**
  • The prevalence of type 2 diabetes in this age cohort has increased significantly to 3/100,000, with the increase driven by increased prevalence rates in both White and Asian populations.
  • Several changes in the epidemiology of pediatric type 2 diabetes noted during COVID-19 were not observed in our cohort.
• **What are the implications of the study?**
  • Factors underpinning changes in disease epidemiology differ across populations and establishment of a National Diabetes Register will facilitate ongoing monitoring.

## 1. Introduction

Studies undertaken prior to the COVID-19 pandemic have emphasized the rising incidence of youth-onset type 2 diabetes with an effective doubling between 2002 and 2018 [1–3]. In the SEARCH study, the annual increase in incidence of type 1 diabetes has remained reasonably steady at approximately 5%, compared to 2% for type 1 diabetes [1, 3]. This observation has been replicated in other cohorts [4–6], although some studies have reported no change [7], however others observed even greater increases [8, 9]. These increases appear to be largely driven by disproportionate increases in higher risk ethnic groups [1, 4, 6, 10]. Consequently, in certain populations a new diagnosis of diabetes in adolescence is now more likely to be type 2 diabetes than type 1 diabetes. It is projected that the prevalence of pediatric type 2 diabetes may increase 700% by 2060, assuming current increases in incidence are sustained [11].

The trajectory of a progressive steady rise in youth-onset type 2 diabetes was altered following the onset of the COVID-19 pandemic. Numerous reports described a dramatic increase in newly diagnosed type 2 diabetes cases [12–17] in addition to alterations, in mode of presentation and disease severity at onset [13, 14, 17]. This rapid increase was of particular concern given that youth-onset type 2 diabetes is ostensibly a more aggressive disease with more rapid beta-cell loss [18] and more rapid progression towards microvascular complications compared to childhood-onset type 1 diabetes [19] or type 2 diabetes with onset in adulthood.

In the Republic of Ireland, the first national study on type 2 diabetes in children and adolescents was undertaken in 2015^20^ and demonstrated a prevalence of 1.2/100,000 in under 16's; lower than many other European countries. At the time, Ireland was projected to have the highest obesity levels in the European Union by 2025. Given, the observations regarding rising incidence of youth-onset type 2 diabetes in the international literature and the changing demographics of our population over time, we aimed to reevaluate the prevalence of type 2 diabetes in the same population as the previous study.

## 2. Methods

Following ethical approval (Reference no: RRECB0323MOG), we conducted a cross-sectional study of children and adolescents currently aged under 16 years with a diagnosis of type 2 diabetes. As previously, approval was granted for the collection of pseudonymized data, to facilitate identification of duplicates. A similar proforma to that used previously [20], with minor alterations to incorporate issues identified during the UK National Pediatric Diabetes Audit (NPDA) [21], was circulated in September 2023 to all clinicians providing care to those aged under 16 with diabetes in all 19 centers in the Republic of Ireland.

Cases were identified from searches of diabetes management software or prospectively maintained electronic spreadsheets. Data from participants' medical records were sought, including demographics, ethnicity, anthropometry, birth history, family history of diabetes, mode of presentation, autoantibody status, associated autoimmune disorders, genetic testing for monogenic diabetes, current and previous treatment, management outcomes and screening for diabetes comorbidities and complications. The diagnosis of diabetes was substantiated according to the International Society for Pediatric and Adolescent Diabetes (ISPAD) criteria: random plasma glucose > 11.1 mmol/L in a patient with hyperglycemic symptoms, fasting plasma glucose ≥ 7.0 mmol/L or HbA1c ≥ 48 mmol/mol (6.5%) or plasma glucose > 11.1 mmol/L at time 120 min during an oral glucose tolerance test (OGTT) [22].

Factors suggesting the presence of type 2 diabetes included elevated body mass index (BMI), evidence of insulin resistance, family history and negative autoantibody status. Overweight and obesity were defined as BMI *z*-scores greater than + 1.3 and + 2.3, respectively. Hypertension was defined according to age- and sex-specific blood pressure centiles. In addition, methods for OGTT and measurement of plasma glucose, insulin, *C*-peptide, HbA1c, low-density lipoprotein (LDL) cholesterol, and diabetes autoantibodies were performed using standard techniques at respective biochemistry laboratories

Data from questionnaires were entered into a Microsoft Excel spreadsheet (Microsoft Corp., Redmond, WA, USA) StatsDirect version 3.3.6 was used for statistical analysis. Descriptive statistics are reported as median (range). Prevalence rates were calculated based on population data obtained from the 2022 Census, published by the Central Statistics office. All prevalence rates are expressed as cases per 100,000 children and adolescents aged under 16 years.

## 3. Results

Responses were obtained from 18 of 19 centers, resulting in 42 completed proformas from 11 centers. The remaining seven centers reported no cases. Following review, 10 cases were excluded as the participants were already aged 16 at the time the study was conducted. The remaining 32 proformas were reviewed and clinical characteristics were deemed consistent with a diagnosis of type 2 diabetes, based on a total population of children and adolescents aged under 16 years in the Republic of Ireland of 1,070,544 the prevalence of pediatric type 2 diabetes was 3/100,000 (95% CI 2–4.2). This represents a significant increase from 1.2/100,000 in 2015 (*p*  < 0.004) [20]. Prevalence estimates by gender and ethnic group are outlined in Table 1. The increase in prevalence from 2015 to 2023 was due to increased prevalence rates in both White and Asian populations as well as an increase in the size of the Asian population in Ireland (Table 2).

In the current cohort, 19 (59%) were girls. The median (range) age at diagnosis was 13 (8–15.3) years. Median duration of diabetes was 1.2 (0–4.9) years. BMI at diagnosis was calculated in 28 (88%) with 14 (50%) being obese. Median BMI *z*-score at diagnosis was + 2.3. The median HbA1c at diagnosis was 85 (48–141) mmol/mol (9.9 (6.5–15.1) %). A family history of type 2 diabetes was present in 25 (78%), either alone 21 (84%) or in combination with type 1 diabetes. Mothers (60%) were the most likely affected relatives, followed by grandmothers (40%) and fathers (36%). A history of diabetes during pregnancy was available in 26 and in 16 (62%) was positive, usually for gestational diabetes (82%).

### 3.1. Diagnosis and Management

Seventeen (53%) had osmotic symptoms and random plasma glucose > 11.1 mmol/L, one of whom had ketoacidosis at diagnosis. Acanthosis nigricans was present in 18 (56%). Diabetes autoantibodies were tested in 26 (81%) and three (12%) were GAD-65-positive and one (4%) was also ZnT8-positive.

Twenty-nine children (91%) were currently on medication. These included 27 (93%) on metformin and 15 (56%) of whom were on monotherapy. Overall, nine (31%) were prescribed insulin and six (21%) were prescribed a glucagon-like-peptide-1 (GLP-1) analog. Twelve (41%) were taking more than one agent concurrently. The current median HbA1c was 46 (34–141) mmol/mol (6.4 (5.3–15.1) %) 16 children (50%) achieved the HbA1c target specified by ISPAD of <48 mmol/mol (6.5%). Median weight change since diagnosis was + 3.2 (−11.6 to + 28.6) kg. Median BMI *z*-score change from diagnosis was −0.12 (−0.85 to + 0.44). BMI *z*-score reduction did not correlate with achievement of HbA1c targets. At the time of the study, five (16%) participants were recategorised as overweight, having been obese at diagnosis, and one (3%) from overweight to obese.

### 3.2. Comorbidities

Blood pressure was assessed at time of diagnosis in 23 children and adolescents (72%). Eight (35%) had stage 1 hypertension, while four (17%) had stage 2 hypertension. Ambulatory blood pressure monitoring was undertaken in three (9%), however only one adolescent was on antihypertensive treatment. Screening for dyslipidaemia was undertaken in 23 (77%). Eleven (48%) of those screened had elevated LDL cholesterol, seven (64%) of whom were above the threshold for initiation of statin therapy. Just one adolescent was prescribed a statin. Eight (35%) had hypertriglyceridemia, including 6 (75%) who also had elevated LDL. Low high-density lipoprotein (HDL) levels (<1 mmol/L) were seen in seven (30%). Screening for complications of diabetes varied with 16 (50%), 18 (56%), and 11 (34%) participants screened for retinopathy, nephropathy and neuropathy, respectively.

### 3.3. Comparison to 2015 Study

The incidence of obesity in the current study was similar to the 2015 study. The HbA1c target specified by ISPAD was achieved by 16 (50%) in the current study, compared to 4 (33%) in the 2015 study, although the difference did not reach statistical significance. The only notable change other than the increase in prevalence was the emergence of use of GLP-1 analogs in six (21%).

## 4. Discussion

Our study demonstrated a significant increase in the prevalence of type 2 diabetes in children and adolescents under 16–3/100,000 from 1.2/100,000 in 2015 using similar methodology. Comparative analysis demonstrated that this was driven by an increased prevalence in White and Asian children in addition to a significant increase in the size of the Asian population under 16 in the intervening period. The point estimate for type 2 diabetes prevalence in the Black population also increased substantially, however the study was underpowered to confirm this statistically. Similarly, no meaningful conclusions can be drawn regarding changes observed in the traveling community population. The overall prevalence of type 2 diabetes, however remains lower than that in the UK NPDA type 2 diabetes spotlight audit (2019–2020) of 4.5 per 100,000. The proportions of the Irish pediatric population within ethnic groups at higher risk of type 2 diabetes are lower than in the UK with respect to both Asian (3.8% vs., 7.5%) and Black (2% vs., 3.3%) children [21], which may account to some degree for the differences.

It is possible that the increased prevalence observed in our study is attributable to increases in type 2 diabetes incidence due to the COVID-19 pandemic. Numerous studies demonstrated increases in incidence ranging from 77% to 293% [12–15, 17] during this period. This was postulated to be the result of greater consumption of ultraprocessed foods, increased screentime and more sedentary behaviors. These were particularly notable during the period of virtual learning [12], while BMI *z*-score in our study cohort was not significantly different from our 2015 study, we do not have up-to-date population-level data on the prevalence of overweight and obesity in children and adolescents in the Republic of Ireland.

The pandemic period also appeared to be associated with a change in the epidemiology of pediatric type 2 diabetes. Several studies reported an increase in incidence in Black [13–15] and a decrease in White children and adolescents [14], however this was not observed in all studies [17]. No significant increases in type 2 diabetes in Asian populations were seen in these studies [12–15], however the proportion of diagnosis in young people of Asian ethnicity was low in some [12]. In our study, no statistically significant increase in the prevalence of type 2 diabetes was observed in Black children and adolescents, with the increase being driven by increases in prevalence in both White and Asian children. Type 2 diabetes in adolescence has continuously been more common in girls, however in some studies during the pandemic, this ratio was reversed [13, 14, 16], although there were indicators in some studies that the trend had started to emerge pre-pandemic. Other studies did not observe this [15, 17], and girls continued to predominate in our cohort. Some US-based studies observed significant increases in the prevalence of obesity [14, 17], however others did not [13, 15]. Similarly, the increase in obesity prevalence was not observed in Germany [16] or indeed in our data. Disparities were also seen in studies regarding differences in mode of presentation. Significant increases in diabetic ketoacidosis (DKA) and hyperglycemic hyperosmolar state (HHS) were observed in several studies [13, 14, 17, 23], however, not universally, and once more, not in our data [15, 16].

Treatment differences compared to the 2015 study were noted with 21% now being prescribed GLP-1 analogs. A recent meta-analysis showed they are associated with substantial reductions in HbA1c, and weight in youth with type 2 diabetes and NICE guidance suggests these agents should be considered [24]. In the US, between 2020 and 2023 there were significant increases in dispensing these medications to young adults (504% in men, 588% in women) [25].

We identified poor compliance with screening for comorbidities and complications of type 2 diabetes. In our study, blood pressure measurement and lipid profiling were undertaken in 72% and 77%, respectively. Hypertension was present in 52%, however just one participant was on antihypertensive treatment. Prevalence of hypertension in the UK NPDA type 2 diabetes spotlight audit was similar (42%) and treatment rates equally low (5.9%), although screening rates for hypertension were higher (95%) [21]. ISPAD guidelines recommend blood pressure measurement at diagnosis and on every clinic visit. Screening rates for dyslipidaemia were similar between our study and the UK NPDA. Almost half of those screened had elevated LDL cholesterol, and among those, two-thirds were above the threshold for statin initiation, however a statin was only prescribed for one eligible participant. Guidelines recommend screening for dyslipidaemia once glycaemic control has been achieved or within 3 months of starting medication irrespective of HbA1c, then annually. Screening for microvascular complications was not conducted in compliance with the guidelines with completion rates ranging from 34% to 56%. Screening for microvascular complications is recommended at diagnosis and annually thereafter.

While, the prevalence of pediatric type 2 diabetes in the Republic of Ireland has increased, the numbers in our study are still relatively small. Our prevalence data assumes that all children with type 2 diabetes are seen in secondary care, however UK data suggests that approximately 20% of those with type 2 diabetes aged under 16 are seen in primary care [26]. There are no data currently available regarding the prevalence of this practice in the Republic of Ireland. Over 50% of the children registered as type 2 diabetes in the Danish Pediatric Diabetes registry, following validation, were patients with type 1 diabetes or monogenic diabetes who were misclassified [27]. Conversely, in a study by the Dutch pediatric surveillance unit, less than two-thirds of the patients believed to have type 2 diabetes, were initially classified as such. The remaining cases were identified among obese children felt to have type 1 diabetes or diabetes of unspecified type [28]. In addition, it is possible that there were additional undiagnosed cases of type 2 diabetes in obese children. Risk factor-based screening as outlined by ISPAD has a relatively low yield, however it could result in the detection in additional cases [22]. The potential limitations outlined are broadly applicable to most studies, such as ours and are unlikely to account for the increase in prevalence seen here. Plans are underway regarding the establishment of a National Diabetes Register which will enable ongoing analysis of disease prevalence and epidemiology.

## Figures and Tables

**Table 1:** Prevalence of type 2 diabetes in under 16's in 2023.

Ethnicity	Cases			Population			Prevalence per 100,000 (95% CI)
	Total	M	F	Total	Boys	Girls	Total
All	32	13	19	1,070,544	547,684	522,859	3 (2–4.2)
White—non–TC	12	6	6	900,845	461,518	439,327	1.3 (0.7–2.3)
Traveler	1	0	1	12,650	6465	6185	7.9 (0.2–44)
Black	6	2	4	21,286	10,736	10,550	28.2 (10.3–61.4)
Asian	13	5	8	40,683	20,569	20,114	32 (17–54.6)

**Table 2:** Comparison of type 2 diabetes prevalence in under 16's between 2023 and 2015 studies.

Ethnicity	2015			2023			
	Cases	Population	Prevalence	Cases	Population	Prevalence	*p*-Value
All	12	1,036,817	1.2 (0.6–2)	32	1,070,544	3 (2–4.2)	<0.004^*∗*^
White—non–TC	4	934,172	0.4 (0.1–1.1)	12	900,845	1.3 (0.7–2.3)	0.038^*∗*^
TC	2	12,442	16.1 (1.9–58.1)	1	12,650	7.9 (0.2–44)	NS
Black	4	30,067	13.3 (3.6–34.1)	6	21,286	28.2 (10.3–61.4)	0.24
Asian	1	23,847	4.2 (0.1–23.4)	13	40,683	32 (17–54.6)	0.021^*∗*^

Abbreviation: TC, traveling community.

*⁣* ^*∗*^ *p*  < 0.05.

## Data Availability

The data that support the findings of this study are available from the corresponding author upon reasonable request.

## Supporting Information

Supporting Information STROBE checklist.
